# Genetically predicted Caspase 8 levels mediates the causal association between CD4+ T cell and breast cancer

**DOI:** 10.3389/fimmu.2024.1410994

**Published:** 2024-09-26

**Authors:** Yanbin Chen, Zequn Zheng, Jinhong Wang, Xifeng Huang, Lei Xie

**Affiliations:** ^1^ Department of Radiology, The First Affiliated Hospital of Shantou University Medical College, Shantou, China; ^2^ Laboratory of Medical Molecular Imaging, The First Affiliated Hospital of Shantou University Medical College, Shantou, China; ^3^ Department of Cardiology, First Affiliated Hospital of Shantou University Medical College, Shantou, Guangdong, China; ^4^ Clinical Research Center, First Affiliated Hospital of Shantou University Medical College, Shantou, Guangdong, China; ^5^ Department of Ultrasound, The First Affiliated Hospital of Shantou University Medical College, Shantou, China

**Keywords:** breast cancer, CD4+T cell, Caspase 8 levels, Mendelian randomization, immune cell

## Abstract

**Background:**

Breast cancer (BC) remains a significant contributor to female mortality globally, with inflammation and the immune system implicated in its pathogenesis. To elucidate potential causal relationships, we evaluated the relationship among 731 immune cell phenotypes and BC be at risk by using Mendelian randomization (MR), while also exploring inflammatory proteins as mediators in this association.

**Methods:**

We obtained immune cell genome-wide association study (GWAS) summary data and 91 inflammatory factors from the GWAS Catalog. BC GWAS data was obtained from the IEU Open GWAS project (ukb-b-16890 for discovery and GCST004988 for validation). We investigated the causal link between immune cells and BC risk by employing a two-sample MR method. Furthermore, we use a two-step MR to quantify the percentage of mediation of immune cell-BC causal effects mediated by inflammatory proteins. To make sure the causal findings were robust, a sensitivity analysis was done.

**Results:**

In both discovery and validation GWAS, a critical inverse correlation between CD4+ T cells and BC risk was found using MR analysis (Discovery: OR, 0.996; P = 0.030. Validation: OR, 0.843; P = 4.09E-07) with Caspase 8 levels mediating 18.9% of the reduced BC risk associated with immune cells(Mediation proportion=a×b/c, Discovery:0.151×-0.005/-0.004 = 18.9%; Validation:0.151×-0.214/-0.171 = 18.9%)

**Conclusion:**

Our study establishes a causal connection linking CD4+ T cells and BC, with Caspase 8 levels partially mediating this relationship. These findings enhance our genetic and molecular comprehension of BC, suggesting potential pathways for future BC immunotherapy drug development.

## Graphical Abstract

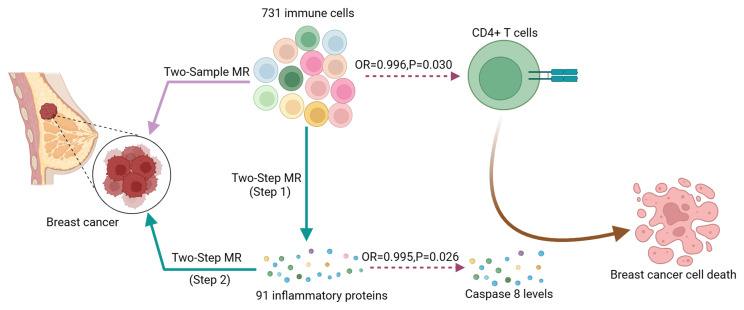

## Introduction

Breast cancer (BC) stands as a formidable health challenge globally, now surpassing lung cancer as the most prevalent malignancy among women (1). Despite its historical perception as having minimal immune involvement, recent advancements in tumor immunology underscore the vital function of immune cells in BC development (2). Additionally, various immune cells and cytokines participate in the BC immune microenvironment, exhibiting both anti-tumor and immune-suppressive functions, and contributing to chronic inflammation linked to BC progression. Recent research indicates that an increase in tumor-infiltrating immune cells, particularly Cytotoxic T Lymphocytes (CTLs), T cells, and B cells, is correlated with a more favorable prognosis in triple-negative breast cancer (TNBC) and high-grade ductal carcinoma *in situ* (DCIS) (3, 4). CTLs are particularly important as they can trigger the death of cancer cells through the Fas/FasL pathway, which leads to apoptosis, or programmed cell death (5). In contrast, pro-inflammatory cytokines such as interleukin-6 (IL-6) have been shown to drive breast cancer progression by creating a tumor-promoting inflammatory environment and inhibiting apoptosis (6, 7). Understanding the immune system’s functionality and associated inflammatory factors is pivotal in designing therapeutic strategies leveraging the immune microenvironment for BC treatment (8). Given the complexity of the immune microenvironment in breast cancer, harnessing the immune system for therapeutic benefit has become a major focus in cancer research (9, 10). Although traditional breast cancer treatments have not typically included immunotherapy (11, 12), emerging evidence suggests that immunotherapeutic approaches can be effective, particularly in subtypes like TNBC, where the presence of immune infiltrates is associated with better outcomes. To establish causality between immune-related factors and breast cancer outcomes, Mendelian randomization (MR) offers a powerful methodological approach (13). By using genetic variants as instrumental variables (IVs), MR can help determine the causal effects of immune-related exposures on breast cancer risk and progression, minimizing the influence of confounding variables and reverse causality that often plague observational studies (14, 15).

Previous research using MR has demonstrated a causal connection between immune cells and ER+ BC (16). However, the expanded genetic information of immune cells and improved MR evaluation models may continually challenge these conclusions. Therefore, based on the availability of a broader spectrum of genetic information on plasma immune cell phenotypes and proteomes, an updated assessment of the causal connection linking BC and immune cells and the role played by inflammatory factors is necessary. Our goal is to employ robust two-sample MR analysis to revisit the relationship of causality among immune cell phenotypes and BC. Additionally, we employ a two-step MR approach to explore the involvement of inflammatory proteins as mediators in this relationship.

## Method

### Study design

**Figure 1:**
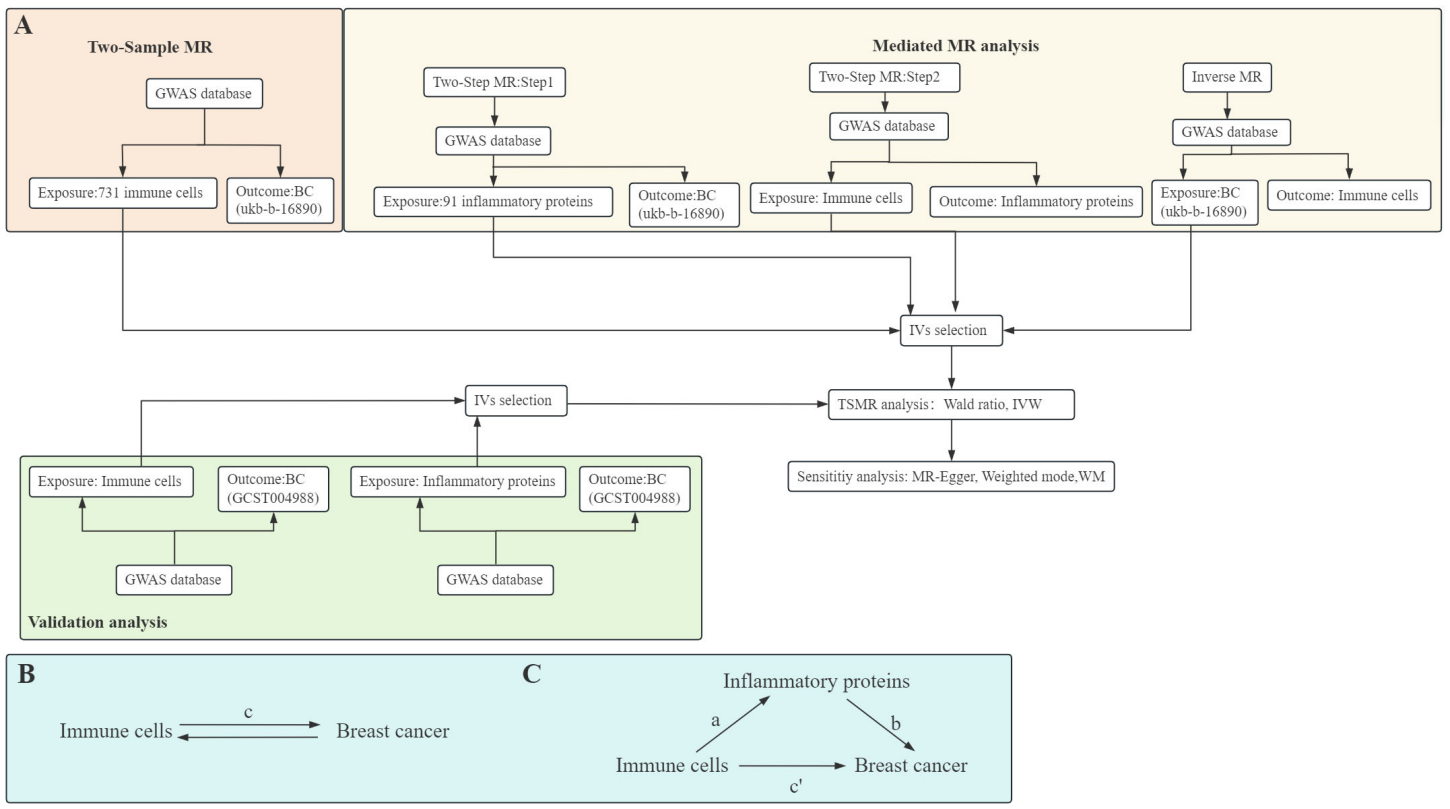
Diagrams illustrate a schematic summary of analysis **(A)**. The overall effect of immune cells on breast cancer (BC) **(B)**, c represents the total effect of immune cells as exposure and BC as outcome. The overall effect is decomposed into **(C)**: (I) indirect effects using the two-step method, a is the total effect of immune cells on inflammatory proteins, b is the effect of inflammatory proteins on BC, and the product method (a × b), and (ii) direct effect (c′=c-a×b). The mediated proportion is calculated as the indirect effect divided by the total effect. TSMR, Two-Sample MR; IVW, Inverse variance weighting; WM, Weighted median.

MR analysis aims to employ variants in genes as IVs to elucidate causal links between exposures and outcomes. It is based on three primary premises: (1) IVs and exposures are associated, (2) IVs don’t depend on confounders, and (3) IVs only have an impact on the outcome by exposure pathways. Initially, to investigate the link of causality among 731 immune cell types and BC risk, we performed two-sample MR. Subsequently, we investigated whether inflammatory factors mediate the link between immune cells and BC risk with a two-step MR methodology. In addition, we used reverse MR analysis to rule out the possible reverse causality between immune cells and BC. Finally, to ensure the robustness of our findings, we employed additional BC GWAS to validate the causal relationship between immune cells and BC and inflammatory factors and BC. A schematic summary of the study is shown in **Figure 1**.

### GWAS summary data sources

Summary data on immune cells and BC were acquired from recently published GWAS datasets.

The immune GWAS data (Study ID: GCST0001391 to GCST0002121) included information on 731 immune phenotypes, categorized into morphological parameters (MP), absolute cells (AC), relative cells (RC), and median fluorescence intensity (MFI) (17). Detailed descriptions are provided in **Supplementary Table S1**.

Additionally, 91 inflammatory factors GWAS were sourced from genome-wide quantitative trait loci (QTL) studies cataloged in HGRI-EBI. This dataset involved 14,824 European participants (Access number: GCST90274758 to GCST90274848), with comprehensive information available in **Supplementary Table S2**.

BC GWAS were obtained from the IEU Open GWAS project. The first BC GWAS investigated 462,933 European individuals (Ncase=10303, Ncontrol=452630) (Study ID: ukb-b-16890). The second BC GWAS investigated 139,274 European individuals (Ncase=76192, Ncontrol= 63082) (Study ID: GCST004988).

### Genetic proxies for causal analysis

We included significantly associated SNPs from genome-wide in our analysis (P < 5×10−8) to be IVs for exposure. Additionally, we define the threshold for linkage disequilibrium (LD) based on pairwise linkage disequilibrium to r2 < 0.001 for the purpose assure IVs independence. When r² > 0.001 in a 10,000 kb span, SNPs that are more associated with additional SNPs or have higher p-values are removed. Furthermore, to confirm the strength of each SNP, we calculated the F-statistic, which assesses the correlation between IVs and exposure. An F-statistic score greater than 10 suggests a strong relationship between IVs and exposure (18). Comprehensive details about SNPs that are used as exposure and outcome, exposure and mediator, as well as mediator and outcome, can be found in **Supplementary Tables S3**, **S6**.

### Statistical analysis

We used R software version 4.3.2 along with the “Two-Sample MR” package for data analysis. Furthermore, we applied various methods including the Wald ratio, inverse variance weighting (IVW), MR-Egger, weighted mode, and weighted median (WM) to evaluate t the link of causality among 731 immune cells, inflammatory proteins, and BC.

### Primary analysis and Sensitivity analysis

Our MR study primarily employed IVW analysis, which relies on outcome variance instead of the term for the intercept in regression, providing robust estimates when directional pleiotropy in the IVs is absent (19). Additionally, when the number of SNPs equals 1, we utilized the Wald ratio as the primary analysis strategy (**Figure 1A**). Furthermore, we performed sensitivity analyses on this MR study using MR-Egger, WM, and weighted mode (20).

### Mediation analysis

To try to determine if inflammatory variables mediate the causal pathway from immune cells to BC outcomes, we employed a two-step MR methodology for mediation analysis (**Figures 1B, C**). This analysis decomposes the overall effect into indirect effects (mediated through the mediator) and direct effects (without mediation) (21). Specifically, the total effect (c in **Figure 1B**) of immune cells on BC is dissected into the direct effect of immune cells on BC (c’ in **Figure 1C**) and immune cells’ indirect effect mediated by the mediator (a × b in **Figure 1C**). We computed the indirect effect’s proportion of the total effect to quantify the portion of the mediated effects.

## Results

### Association of immune cells with BC

**Figure 2:**
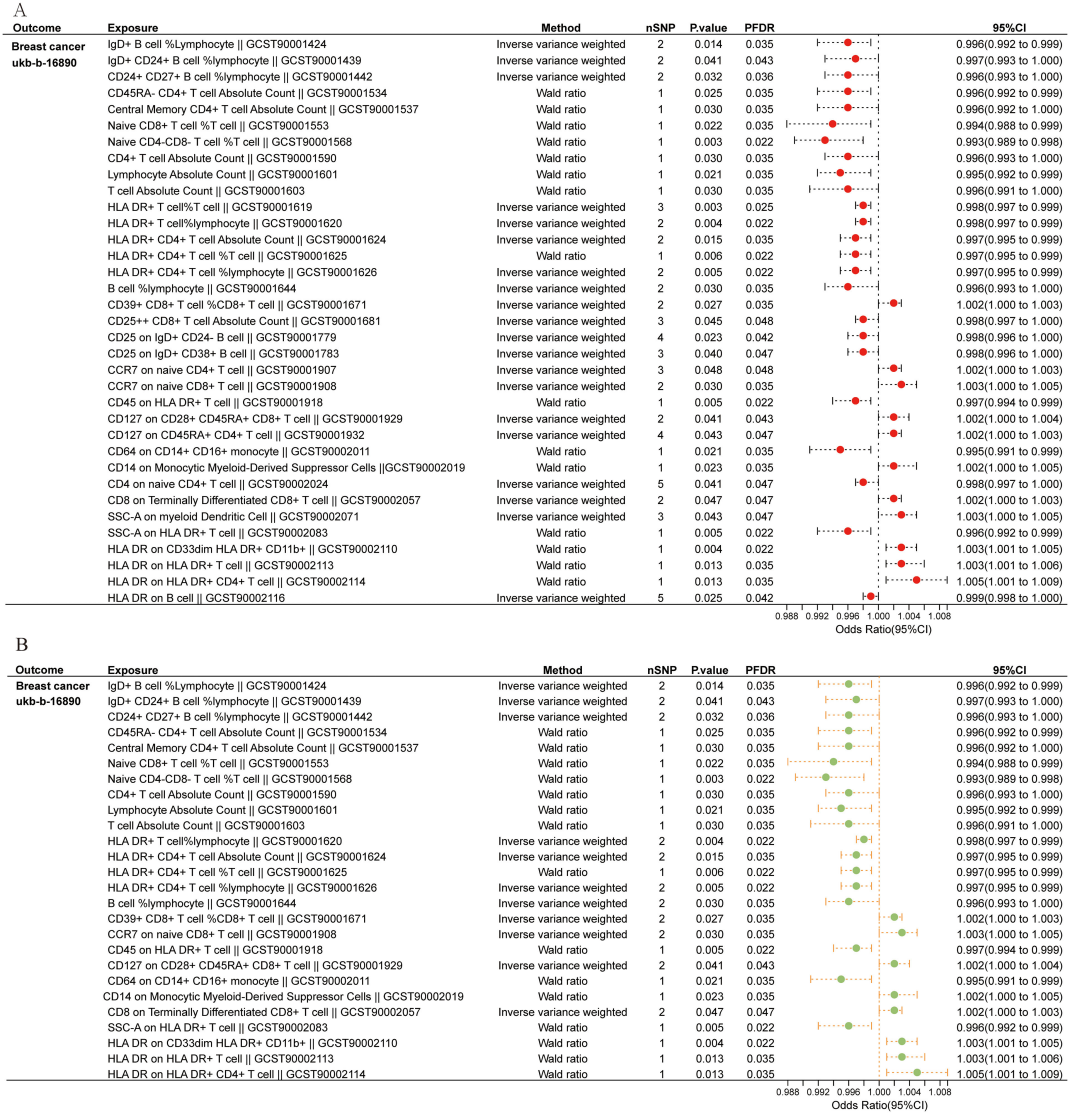
Immune cells causally linked to breast cancer. **(A)** Forest plot depicted 35 types of immune cells causally linked to breast cancer. **(B)** Robust MR result exhibited 26 immune cell types associated with breast cancer.

Initially, we examined the connection between 731 immune cell types and BC. **Supplementary Table S4** provides detailed information on MR estimations for immune cells and BC. The results indicated that among the 731 immune cell types, 35 immune cell types were found to have a causal relationship with BC (**Figure 2A** **).** Subsequently, we conducted sensitivity analyses on these 35 immune cell phenotypes potentially causally related to BC through MR-Egger, WM, and weighted mode methods. Robust MR results supported a total of 26 immune cell phenotypes associated with the occurrence of BC (**Figure 2B**, **Supplementary Table S5**). The MR analysis identified 26 immune cell phenotypes associated with BC occurrence. Of these, 18 were protective, including various T cell and B cell types, while 8 were risk factors, primarily T cell groups. Among them, IgD+ B cells (OR, 0.996; P = 0.014), CD4+ T cells (OR, 0.996; P = 0.030), and HLADR+ CD4+ T cells (OR, 0.997; P = 0.006) indicated a negative association with the occurrence of BC. Conversely, CD39+ CD8+ T cells (OR, 1.002; P = 0.027) and CCR7 on naive CD8+ T cells (OR, 1.003; P = 0.030) were positively linked to the occurrence of BC.

### Association of inflammatory proteins with BC

**Figure 3:**
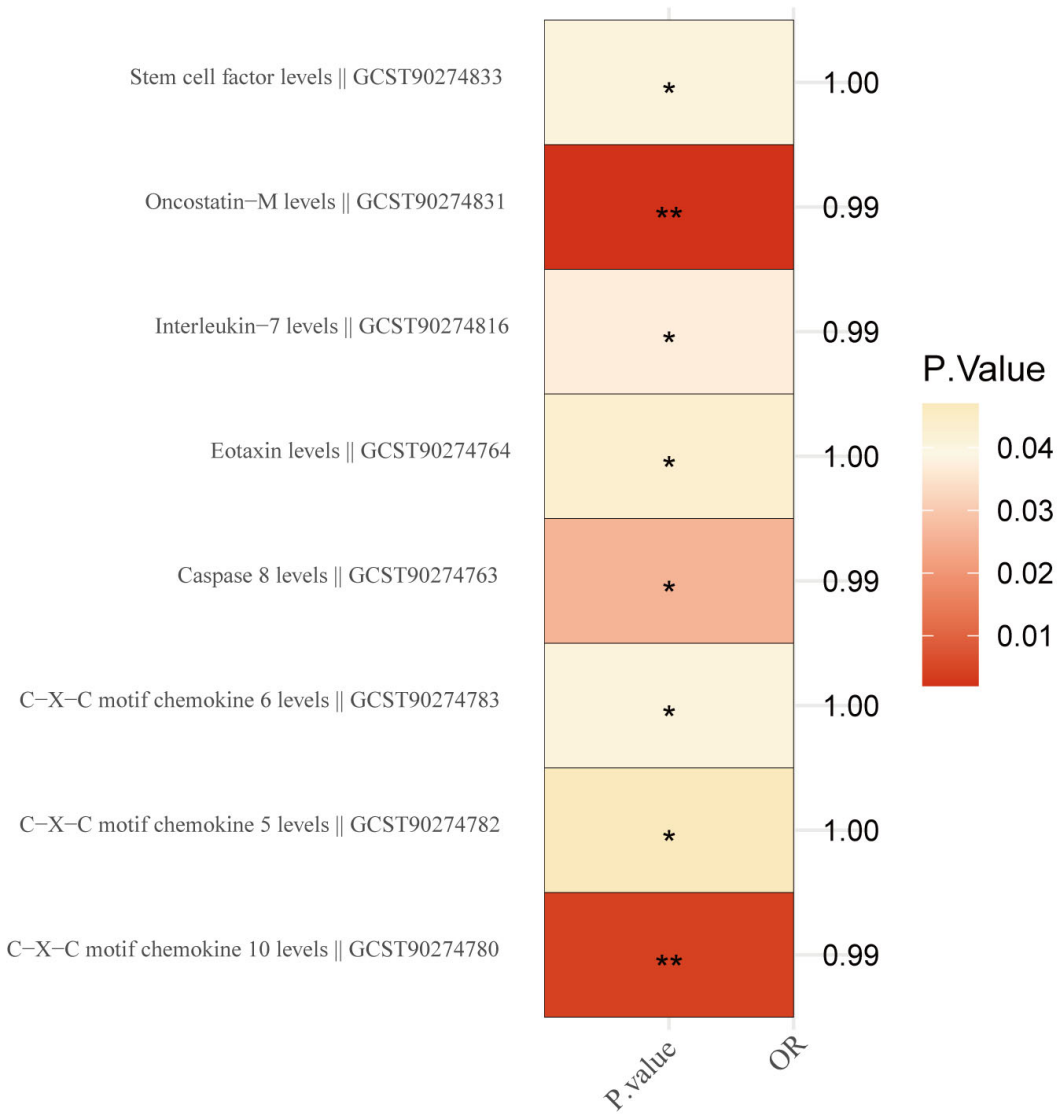
Inflammation proteins causally linked to breast cancer. '*' indicates that P. value <0.05, while the symbol '**' indicates that P. value <0.01.

We investigated the possible causality link between 91 inflammatory proteins and BC. Detailed information on MR estimates on 91 inflammatory proteins and BC are provided in **Supplementary Table S7**. The results revealed that 8 out of the 91 inflammatory factors were found to have a causal relationship with BC (**Figure 3**). Among these, 5 inflammatory factors were identified as protective factors (Interleukin-7 levels: OR,0.994; P = 0.037; Oncostatin-M levels: OR,0.994; P=0.002; Caspase 8 levels: OR, 0.995; P = 0.026; C-X-C motif chemokine 10 levels: OR,0.995; P = 0.004; C-X-C motif chemokine 5 levels: OR,0.999; P=0.047), while 3 were identified as risk factors(Eotaxin levels: OR, 1.002; P = 0.043; C-X-C motif chemokine 6 levels: OR, 1.002; P = 0.041; Stem cell factor levels: OR, 1.002; P = 0.041). Robust MR results for the aforementioned inflammatory factor phenotypes showed that 2 inflammatory factors Caspase 8 levels (OR, 0.995; P = 0.026, PFDR = 0.047) and Interleukin-7 levels (OR, 0.994; P = 0.037, PFDR = 0.047 causally related to BC (**Supplementary Table S8**).

### Mediated effect for inflammatory proteins

**Figure 4:**
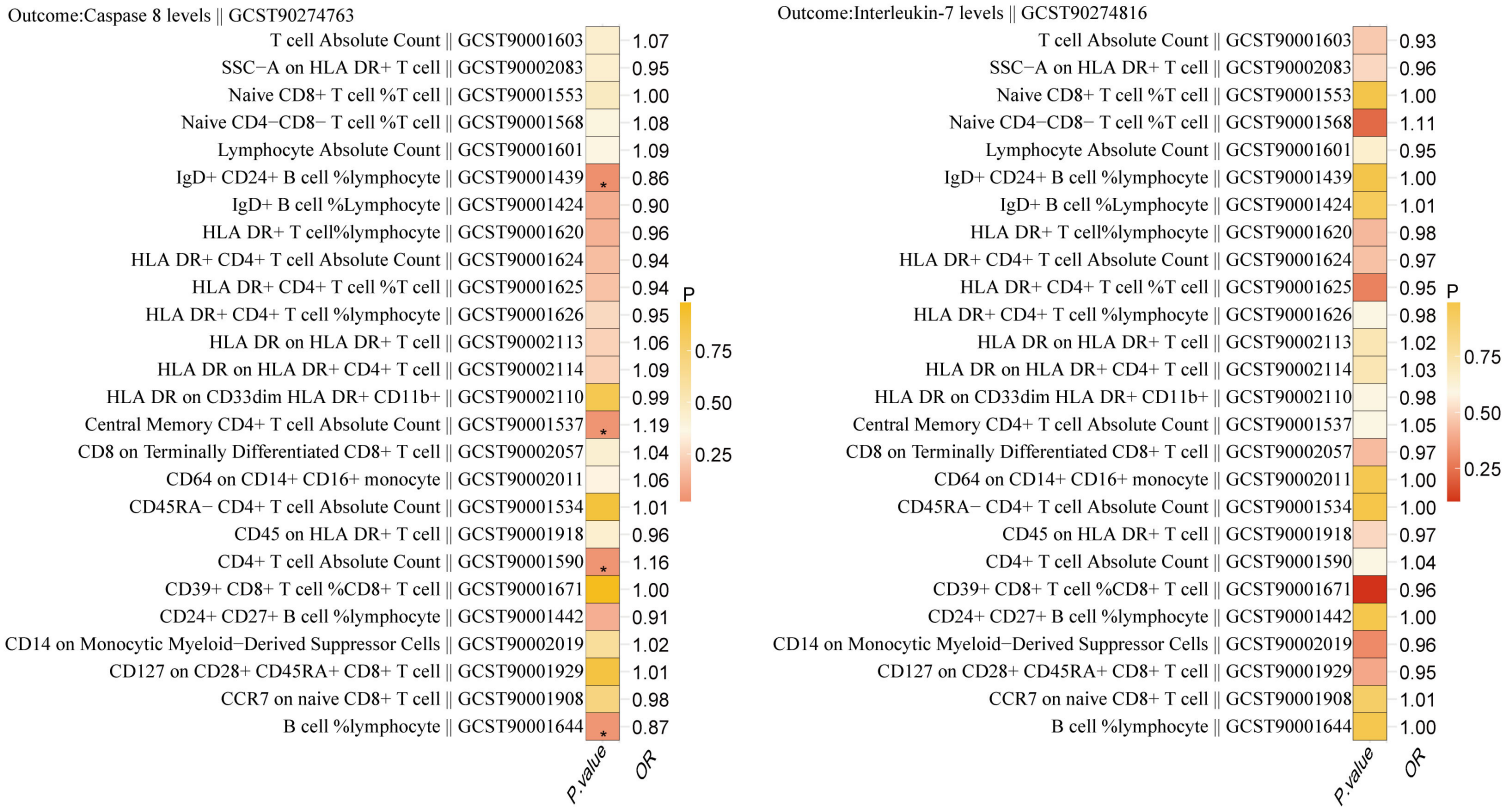
Relationship between 26 immune cells associated with breast cancer and the inflammatory proteins Caspase 8 levels and Interleukin-7 levels. '*' also indicates that P. value <0.05.

Using two-step MR, we examined the causal association between 26 immune cell types significantly associated with BC and Caspase 8 levels and Interleukin-7 levels. The results indicated that 4 immune cell types have a causal relationship with the inflammatory protein Caspase 8 levels (**Figure 4**). They are B cell (OR, 0.874; P = 0.041),IgD+ CD24+ B cell (OR, 0.858; P = 0.023),Central Memory CD4+ T cell Absolute Count (OR, 1.188; P = 0.036),CD4+ T cell Absolute Count (OR, 1.163; P = 0.036).

### Reverse MR analysis

We conducted reverse MR analysis on the aforementioned four immune cell phenotypes to investigate their reverse causal relationships with BC. Detailed result information was provided in **Supplementary Table S9**. The results indicated that none of the four immune cells exhibit reverse causal relationships with BC. Specifically, for CD4+ T cells and BC, the P-value is 0.641.

### Validation analysis

We used another BC GWAS (Study ID: GCST004988) to validate the relationship between the aforementioned four immune cells and BC. Detailed result information is shown in **Supplementary Table S10**. The results showed that CD4+ T cells were causally associated with BC (OR=0.843; P=4.09×10-7), whereas B cells were not causally associated with BC (OR=0.987; P=0.846). Additionally, we used this BC GWAS to validate the causal relationships between the aforementioned two inflammatory proteins and BC. Detailed information is provided in **Supplementary Table S11**. The results indicate that Caspase 8 levels are causally associated with BC (OR=0.807; P=4.88×10-6), while Interleukin-7 levels do not exhibit a causal relationship with BC (OR=1.074; P=0.245).

**Figure 5:**
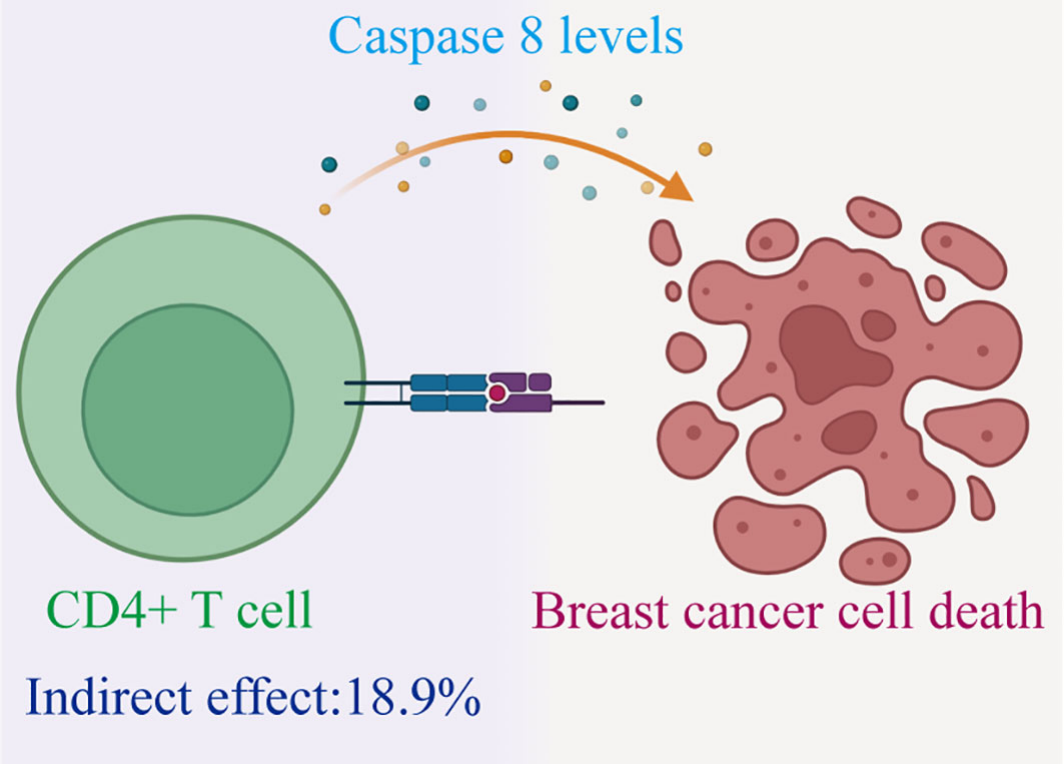
Schematic diagram of the Caspase 8 levels mediation effect.

Therefore, we have identified Caspase 8 levels serve as a potential mediator within the pathway that connects immune cells to BC. Specially, we found that an increase in CD4+ T cells has a relationship with a higher degree of Caspase 8, and higher Caspase 8 levels are associated with a reduced risk of BC (**Figure 5**). Our study indicates that Caspase 8 levels account for 18.9% of the reduction in BC risk associated with immune cells (mediation proportion=a×b/c, Discovery:0.151×-0.005/-0.004 = 18.9%; Validation:0.151×-0.214/-0.171 = 18.9%).

## Discussion

We examined the causal links among 731 immune cell characteristics, inflammatory proteomes, and BC using extensive publicly accessible genetic datasets. Additionally, we investigated the potential mediation role of inflammatory factors to better understand the underlying mechanisms governing the interplay between immune cells and BC. According to our study, 26 immune cell types and 2 inflammatory proteins were discovered to have essential causal interactions with BC. Notably, CD4+ T cells exhibited a negative correlation with BC through their association with Caspase 8 levels.

Our study revealed a positive association between CCR7 expression on naive CD8+ T cells and the occurrence of BC. CCR7, a chemokine receptor predominantly found on immune cells which includes T cells, B cells, and dendritic cells, is essential to regulate cell migration and positioning within lymphoid tissues. Additionally, increased expression of HLA-DR on both HLA-DR+ T cells and HLA-DR+ CD4+ T cells was observed in BC cases. Professional antigen-presenting cells have a class II major histocompatibility complex (MHC) molecule called HLA-DR. This molecule is essential for presenting antigenic peptides to CD4+ helper T cells, which in turn indicates T cell activation. The upregulation of HLA-DR typically occurs within 24-48 hours post-activation, accompanied by heightened IFN-γ production (22–26). Recent studies have shown elevated levels of HLA-DR on CD8+ T cells in various types of cancer (27, 28). These activated CD8+ T cells execute target cell apoptosis through cytolytic (cell-mediated cytotoxicity) and non-cytolytic (cytokine production) mechanisms. The former involves the release of cytotoxic molecules like perforin and granzyme B, while the latter entails the secretion of key cytokines like IL-2, TNF-α, and IFN-γ, crucial for antiviral and inflammatory responses (29)^-^ (30). Interestingly, our findings of an association between elevated CCR7 and HLA-DR expression levels on T cells and BC occurrence contrast with previous studies. This inconsistency underscores the need for further research to unravel the underlying mechanisms driving BC development.

Lymphocytes, which include CD19+ B cells, CD3+ T cells, CD4+ T cells, CD8+ T cells, CD3+CD4−CD8− T cells, and Nature Killer cells (NK cells), are essential for the immunological response in the human body. CD3+ T cells encompass the total T lymphocyte population, including CD4+ and CD8+ T cells, pivotal in cellular immunity. CD4+ T cells, central to immune function, release anti-tumor cytokines and stimulate more immune effectors (31). Subsets such as Th1, Th2, and regulatory T cells (Treg) are formed by further differentiation of CD4+ T cells. Th2 and Treg cells have an immunosuppression impact, which promotes tumor growth, whereas Th1 cells drive responses against tumors. Numerous immune cell subpopulations, such as B cells, T cells, eosinophils, neutrophils, dendritic cells, basophils, monocytes, mast cells, macrophages, and NK cells, are found in the normal breast ductal layer. These cells confer continuous protection to mammary epithelium against pathogens and eliminate transformed cells during breast development (32, 33). However, tumor cells employ immune evasion strategies by secreting suppressive factors that hinder immune cell differentiation and proliferation. Reduced immune cell levels and altered CD4+/CD8+ ratios have been observed in various cancers, indicating immune evasion (34, 35). Our study uncovered a negative association between CD4+ T cells and BC incidence, suggesting a potential role of decreased CD4+ T cell numbers in BC development. This finding highlights the intricate interplay between immune cells and BC pathogenesis, warranting further investigation into immune-mediated mechanisms in BC.

Importantly, we identified Caspase 8 as a potential mediator in the CD4+ T cells-BC causal pathway. The family of cysteine proteases known as Caspases regulates the beginning and completion of apoptosis by activating proteins via intricate direct and feedback mechanisms (36). In the absence of apoptotic signals, caspase proteins remain inactive as pro-caspases. To date, there are 14 known mammalian caspases, that are categorized based on their functions into initiator (e.g., caspase-2, -8, -9, and -10), effector (e.g., caspase-3, -6, and -7), and cytokine maturation and inflammation-related caspases (e.g., caspase-1, -4, and -5) (36, 37). Caspase-8, a pivotal member, is instrumental in inducing cell apoptosis triggered by death receptor activation. It is drawn to the death-inducing signaling complex (DISC) mediated by CD95 (Fas/APO-1) and ultimately causes cell apoptosis. Previous research, including that by YANG et al., has suggested that caspase downregulation or deficiency could contribute to BC development (38). Our study corroborated these findings by revealing an association between Caspase 8 levels and a reduced risk of BC occurrence.

Previous studies have shown that before the clinical diagnosis of BC, circulatory leukocyte composition has changed (39). Also, differences in blood leukocyte composition between diagnosed but untreated BC patients and women without BC have been observed (40, 41). Identifying changes that may occur in the immune system of patients with BC will help us understand which immune cells can serve as effective targets for immunotherapies. Our study suggests an association between CD4+ T cells and reduced risk of BC via Caspase 8, and that activation of CD4+ T cells is critical in coordinating and maintaining initial immune response and generating anti-tumor responses such as immune memory (42), which may be an effective potential target for BC immunotherapy.

Although we applied the latest available large-scale genetic data for the phenotypes of interest, our study has certain limitations. Primarily, the fact that our results are based on a European database may constrain their applicability to non-European ethnic groups. To address this limitation, establishing databases representing diverse populations will be crucial for comprehensive insights into immune-related phenotypes across different ethnicities. Additionally, future research endeavors should aim to elucidate the dynamic alterations in host immune status and inflammation throughout the disease continuum. Stratifying patients according to their immune profiles may emerge as a requisite step in tailoring personalized treatment regimens. Moreover, there is a pressing need for investigations focusing on refining immune subtypes and characterizing the body’s inflammatory responses within the immune microenvironment. Such endeavors hold promise for facilitating more targeted and precise therapeutic interventions in the management of various diseases.

## Conclusion

We observed a significant link between immune cells and BC, notably a reduced BC risk associated with CD4+ T cells potentially via Caspase 8. These findings have implications for future research, particularly in BC vaccine and immunotherapy drug development. However, further investigation is warranted to fully understand this association.

## Data Availability

The original contributions presented in the study are included in the article/**Supplementary Material**. Further inquiries can be directed to the corresponding author.

## Supplementary material

Supplementary Table 1 Detailed information on the genome-wide association studies (GWAS) of 731 immune cells.

Supplementary Table 2 Detailed information on the GWAS of 91 inflammation proteins.

Supplementary Table 3 Genetic instrumental variables for immune cells.

Supplementary Table 4 Mendelian randomization estimates of immune cells and BC.

Supplementary Table 5 Sensitivity analyses on 35 immune cell phenotypes potentially causally related to BC.

Supplementary Table 6 Genetic instrumental variables of inflammatory proteomes.

Supplementary Table 7 Mendelian randomization estimates of 91 inflammatory proteins and BC.

Supplementary Table 8 Sensitivity analyses on 6 inflammatory proteins potentially causally related to BC.

Supplementary Table 9 Reverse MR analysis between 4 immune cell phenotypes and BC.

Supplementary Table 10 Validation analysis of 4 immune cell phenotypes and BC.

Supplementary Table 11 Validation analysis of 2 inflammatory proteins and BC.
